# Causal relationship between schizophrenia and five types of dementia: A bidirectional two-sample Mendelian randomization study

**DOI:** 10.1371/journal.pone.0322752

**Published:** 2025-05-08

**Authors:** Feng Tao, Shizhe Deng, Bifang Zhuo, Jingyue Liu, Xuan Liang, Jiangwei Shi, Zhihong Meng

**Affiliations:** 1 First Teaching Hospital of Tianjin University of Traditional Chinese Medicine, Tianjin, China; 2 National Clinical Research Center for Chinese Medicine Acupuncture and Moxibustion, Tianjin, China; Edith Cowan University, AUSTRALIA

## Abstract

**Background:**

Although observational research indicates an association between schizophrenia and dementia, it is unclear whether the two are causally related. In order to examine the causal relationship between schizophrenia and five types of dementia (all-cause dementia, Alzheimer’s disease, vascular dementia, frontotemporal dementia, and dementia with Lewy bodies), we performed a bidirectional two-sample Mendelian randomization analysis.

**Methods:**

In this study, pooled statistics of schizophrenia and dementia were obtained from the Large-scale genome-wide association study (GWAS) in individuals of European ancestry. Inverse variance weighted (IVW) was the primary statistical approach used in this Mendelian randomization, to further support our findings, we also used MR-Egger, weighted median, and cML-MA. We also used a number of sensitivity analyses to evaluate pleiotropy and heterogeneity.

**Results:**

In the study of the effect of schizophrenia on dementia, findings from the IVW analysis suggested that schizophrenia is associated with an increased risk of all-cause dementia (OR = 1.065, 95%CI: 1.027 ～ 1.104, *P* = 0.001, FDR-corrected *P* = 0.003), Alzheimer’s disease (OR = 1.029, 95%CI: 1.003 ～ 1.054, *P* = 0.027, FDR-corrected *P* = 0.045), and vascular dementia (OR = 1.106, 95%CI: 1.023 ～ 1.197, *P* = 0.012, FDR-corrected *P* = 0.029). In the study of the effect of dementia on schizophrenia, no form of dementia assessed in this study was found to be a risk factor for schizophrenia.

**Conclusion:**

Our findings suggest that schizophrenia may be a risk factor for all-cause dementia, Alzheimer’s disease, and vascular dementia, but no dementia of any kind was found to be a risk factor for schizophrenia. Our study provides insights into the potential genetic relationship between schizophrenia and dementia.

## Introduction

Dementia is a syndrome caused by various brain diseases (such as neurodegenerative diseases, cerebrovascular lesions, and others), and is characterized by progressive cognitive and functional decline. It is one of the leading causes of disability worldwide [1]. Alzheimer’s disease (AD) is the most common form of dementia, accounting for approximately 60–70% of cases, while other major types include vascular dementia (VaD), frontotemporal dementia (FTD), and dementia with Lewy bodies (DLB) [2,3]. Recent projections indicate that by 2050, the number of patients living with dementia worldwide will rise to 139 million, which will have profound impacts on healthcare systems and socioeconomic [4]. However, the pathogenesis of dementia is complex, and no curative treatments are currently available. Assessing the shared genetics between dementia and other diseases (comorbidities) may contribute to the development of potential therapeutic strategies [5].

Schizophrenia is a severe psychiatric disorder characterized by symptoms such as hallucinations, delusions, disorganized thinking, and emotional blunting, which are often accompanied by cognitive decline [6]. Emerging evidence suggests that dementia and schizophrenia may share a common genetic basis [7,8]. Furthermore, multiple studies have reported an association between schizophrenia and dementia. For example, A meta-analysis of cohort studies indicated that patients living with schizophrenia have a higher risk of developing dementia compared to those without schizophrenia (relative risk [RR] = 2.29; 95% confidence interval [CI]: 1.35 ~ 3.88) [9]. A cohort study in Danish, based on 2.8 million people, conducted follow-up for 18 years and found that the risk of all-cause dementia (ACD) in patients living with schizophrenia is more than twice that of the general population (incidence rate ratio [IRR]= 2.13; 95% CI: 2.00–2.27) [10]. Another cohort study revealed that patients living with schizophrenia had the high risk of developing AD (adjusted hazard ratio [aHR] = 2.10; 95% CI = 1.88 ± 3.86; p < 0.001) and VaD (aHR = 1.67; 95% CI = 1.27 ± 2.12; p < 0.001) [11]. In addition, Velakoulis D et al. reported in a review of clinical cases that patients living with early-onset FTD might be diagnosed with schizophrenia years prior to a dementia diagnosis [12]. A cross-sectional investigation has shown the presence of very late-onset schizophrenia-like psychosis as a distinguishing feature of prodromal DLB [13]. However, the conclusions of observational studies are easily influenced by confounding factors and reverse causality, making them insufficient to provide support for causality.

Mendelian randomization (MR) is a powerful epidemiological method that leverages genetic variants as instrumental variables (IVs) to infer causality [14]. Since genetic variants are randomly assigned at conception, MR minimizes the influence of confounding factors and reverse causality [15]. The genome-wide association study (GWAS) database is a widely used open-access resource that provides information on diseases and their genetic associations. In the past, three MR studies have explored the causal relationship between multiple mental disorders and AD [16–18]. However, past studies have focused solely on AD, lacking an exploration of the causal relationships between schizophrenia and other dementia subtypes. Moreover, the GWAS data for both schizophrenia and AD have been extensively updated. Therefore, it is necessary to leverage larger and more recent GWAS datasets to further investigate the causal relationships between schizophrenia and more dementia subtypes, providing multidimensional insights for MR study and enhancing the reliability of causal inference.

In this study, we conducted a bidirectional two-sample MR analysis based on the latest large-scale GWAS summary statistics to evaluate the potential causal relationships between schizophrenia and five types of dementia (ACD, AD, VaD, FTD, and DLB).

## Methodology

### Study design

**Fig 1:**
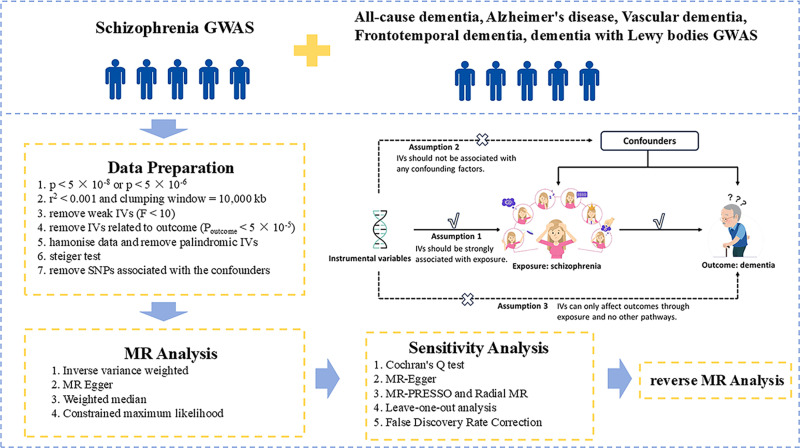
Schematic diagram of the Mendelian randomization Analysis. MR, Mendelian randomization; GWAS, Genome-Wide Association Study; SNPs, single nucleotide polymorphisms; IVs, instrumental variables.

We conducted an MR study based on a publicly available GWAS dataset. The original GWAS already had patient informed consent and ethical approvals. Therefore, no additional ethical approval was needed for this study. The schematic diagram of the MR analysis is shown in Fig 1.

The MR study must adhere to the following three key assumptions: 1) the assumption of relevance: IVs should be strongly associated with exposure; 2) the assumption of independence: IVs should not be associated with any confounding factors; 3) the assumption of exclusivity: IVs can only affect outcomes through exposure and no other pathways. This study is reported according to the STROBE-MR statement (S1 File).

### Data sources

The GWAS pooled data for schizophrenia was obtained from the Psychiatric Genomics Consortium (PGC), which includes 53,386 cases of schizophrenia and 77,258 controls [19]. The PGC represents the largest consortium in the history of psychiatry which translate family history risk factors into biologically, clinically, and therapeutically meaningful insights. Over the past decade, it has advanced our understanding of common psychiatric disorders [20].

The GWAS dataset for ACD was collected from the FinnGen Consortium, which includes 18,216 cases of ACD and 330,460 controls. The GWAS dataset for AD was derived from stage I of the European Alzheimer’s and Dementia Biobank (EADB), including 85,934 cases (39,106 clinically diagnosed cases and 46,828 proxy cases) and 401,577 controls [21]. The GWAS dataset for VaD was also collected from the FinnGen Consortium, which includes 3,116 cases of VaD and 433,066 controls. The GWAS dataset for FTD was derived from research comprising 3024 individuals (515 cases of FTD and 2509 controls) [22]. The GWAS summary statistics for DLB were acquired from a study done by Ruth Chia et al. This study comprised 6,618 individuals, consisting of 2,591 cases of DLB and 4,027 controls [23]. In addition, the GWAS summary data are detailed in Table 1.

**Table 1:** Data sources used for the Mendelian randomization analysis.

Trait	Population	Authors and Publish Time	Case	Control	GWAS ID	Websource
Schizophrenia	European	Trubetskoy V et al.,2022	53,386	77,258	schizophrenia	https://pgc.unc.edu/
All-cause dementia	European	FinnGen (R11),2024	18,216	330,460	KRA_PSY_DEMENTIA_EXMORE	www.finngen.fi/en
Alzheimer’s disease	European	Bellenguez C et al.,2022	85,934	401,577	GCST90027158	10.1038/s41588-022–01024-z
Vascular dementia	European	FinnGen (R11),2024	3,116	433,066	F5_VASCDEM	www.finngen.fi/en
Frontotemporal dementia	European	Van Deerlin VM et al.,2010	515	2,509	ieu-b-43	10.1038/ng.536
Dementia with Lewy bodies	European	Chia R et al.,2021	2,591	4,027	GCST90001390	10.1038/s41588-021-00785-3

### Selection of instrumental variables

To fulfil the three key assumptions, we have developed a strict protocol for selecting IVs. First, for the schizophrenia-dementia study, only single-nucleotide polymorphisms (SNPs) that showed significant associations (p < 5 × 10^−8^) with exposure were selected as IVs. However, for the dementia-schizophrenia study, only a few SNPs met this stringent threshold (p < 5 × 10^−8^), so we used a lower standard (p < 5 × 10^−6^). In the past MR studies, such thresholds are commonly used when SNPs counts are limited [24,25]. Second, we applied linkage disequilibrium (LD) clumping to identify independent SNPs meeting the specified criteria (r^2^ < 0.001 within a clumping distance of 10,000 kb). Third, to minimize the influence of weak instrument bias, we removed SNPs having F-values below 10. The F-value was computed using the following formula:


$$F=R^{2}\frac{\left(N-2\right)}{\left(1-R^{2}\right)}$$

where N is the sample size and R^2^ represents the proportion of variance in the exposure explained by genetic variants. The specific formula used to calculate R^2^ is as follows:


$$R^{2}\;=\;2\;\times \;EAF\;\times \;(1\;-\;EAF)\;\times \;\beta^{2}$$

where β represents the effect estimate of the genetic variant in the exposure GWAS, and EAF is the effect allele frequency [26]. Fourth, we excluded SNPs that showed a strong association with the outcome (p < 5 × 10 ⁻ ⁵). Fifth, we harmonized and integrated the selected datasets of exposure and outcome to guarantee that the effect alleles belonged to the same allele, and eliminated palindromic SNPs. Sixth, we conducted a Steiger test to filter out reverse causal SNPs, excluding those that tested in the “FALSE” direction [27]. Finally, we queried the phenotype information associated with SNPs using the NHGRI-EBI Catalog (https://www.ebi.ac.uk/gwas/) and removed SNPs associated with potential confounders.

### Statistical analysis

This MR study utilized four statistical approaches: Inverse variance weighted (IVW), MR Egger, Weighted median (WM) and cML-MA.

Among these, the IVW is the primary statistical approach for our MR study. It assumes that IVs affect the outcome exclusively through the specific exposure and employs the meta-analysis approach to integrate Wald ratios of individual SNPs. In the absence of horizontal pleiotropy and heterogeneity, IVW linear regression provides unbiased causal estimates [28]. The WM is expected to offer reliable causal estimates even if up to 50% of the IVs are invalid [29]. The MR-Egger can assess whether genetic variants have pleiotropic effects on the outcome and provide a consistent estimate of the causal effect [30]. However, the MR-Egger method often has bias and low efficacy, even if its appropriateness for MR in the presence of horizontal pleiotropy. The cML-MA technique is a novel methodology. We employed the cML-MA approach to tackle correlated and uncorrelated pleiotropic effects, and it was believed to be more potent than MR-Egger, with superior type I error control, and could reach greater statistical efficacy [31].

### Sensitivity Analysis

We used several methods for sensitivity analysis. Both the IVW and MR-Egger were utilized in the execution of the Cochran’s Q test [28], which was designed to assess the heterogeneity in causality (p < 0.05 indicates presence of heterogeneity). Then, the MR-Egger intercept [30] and MR-Pleiotropy Residual Sum and Outlier method (MR-PRESSO) Global Test [32] were employed to assess pleiotropy (p < 0.05 indicates presence of pleiotropy). If there is heterogeneity or pleiotropy, we detect potential outliers using the MR-PRESSO [32] and Radial MR [33] methods. If potential outliers were found, we discarded them and re-executed the MR analysis. The leave-one-out analysis was also carried out in our MR study. This technique consisted of eliminating one SNP at a moment; if influential SNPs were present, we treated the results with caution; otherwise, the results were considered robust.

We applied the Benjamini Hochberg technique for FDR correction in order to lower the possibility of false positives [34]. There is a significant causal relationship when the FDR corrected P < 0.05 and the original P < 0.05 [35]. RStudio was utilized to conduct MR analysis, with TwoSample MR (version 0.5.8) being the R package utilized.

## Results

### Selection of instrumental variables

In the schizophrenia-dementia study, we excluded SNPs with LD, removed SNPs strongly associated with the outcome or were palindromic (S1 Table), and retained SNPs with F-value > 10. We then conducted a Steiger test to exclude SNPs likely to exhibit reverse causality (S1 Table). Then we removed SNPs related to potential confounding factors (smoking, drinking, hypertension, diabetes, body mass index [36–40]) (S1 Table). Finally, we employed MR-PRESSO and Radial MR methods to identify and exclude any potential outliers (S1 Table). The remaining SNPs were then used in MR analysis, with detailed information provided in S2 Table.

In the dementia-schizophrenia study, we applied the same methods to select IVs and found no SNP associated with confounders for schizophrenia. The ultimate SNPs employed in the MR analysis are enumerated in S2 Table.

### The causal effect of schizophrenia on dementia

**Fig 2:**
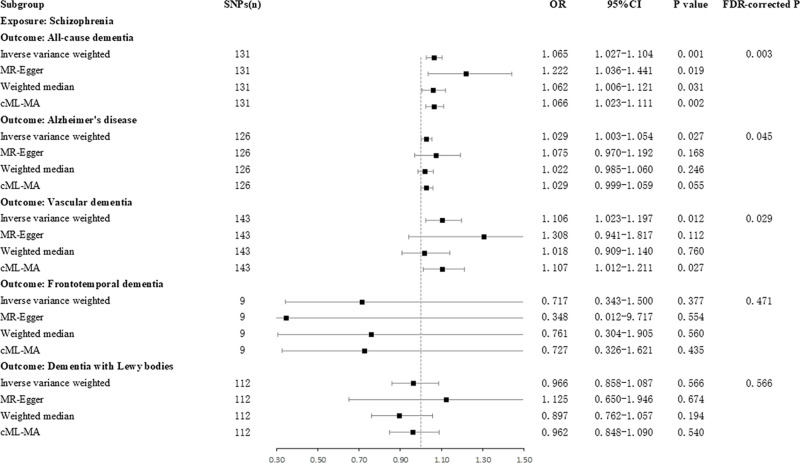
The causal effect of schizophrenia on dementia. SNPs, single nucleotide polymorphisms; OR, odds ratio; CI, confidence interval.

Results from the IVW method revealed that schizophrenia is associated with an increased risk of ACD (odds ratio [OR]=1.065, 95%CI: 1.027 ～ 1.104, *P* = 0.001, FDR-corrected *P* = 0.003), AD (OR = 1.029, 95%CI: 1.003 ～ 1.054, *P* = 0.027, FDR-corrected *P* = 0.045), and VaD (OR = 1.106, 95%CI: 1.023 ～ 1.197, *P* = 0.012, FDR-corrected *P* = 0.029). In contrast, no causal association was found between schizophrenia and FTD (OR = 0.717, 95%CI: 0.343 ～ 1.500, *P* = 0.377, FDR-corrected *P* = 0.471) or DLB (OR = 0.966, 95%CI: 0.858 ～ 1.087, *P* = 0.566, FDR-corrected *P* = 0.566) (Fig 2).

The associations between schizophrenia and ACD, AD, and VaD did not show any signs of heterogeneity (P > 0.05) or pleiotropy (P > 0.05), according to sensitivity analysis (Table 2, S2 File). The funnel plot displayed a symmetrical distribution of SNPs (S3 File). The leave-one-out analysis displayed consistent results upon the exclusion of individual SNPs, with no single SNP exerting disproportionate influence on the overall estimate, thereby demonstrating the results robustness (S4 File).

**Table 2:** Sensitivity analysis results.

	Heterogeneity		Pleiotropy	
Outcome	P (MR Egger)	P (Inverse variance weighted)	P (Egger intercept)	P (MR-PRESSO Global Test)
All-cause dementia	0.729	0.687	0.096	0.699
Alzheimer’s disease	0.941	0.943	0.382	0.952
Vascular dementia	0.229	0.227	0.306	0.220

### The causal effect of dementia on schizophrenia

**Fig 3:**
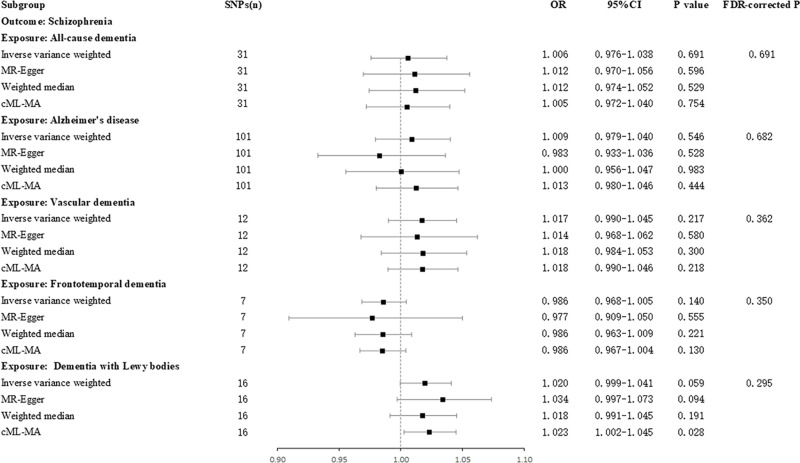
The causal effect of dementia on schizophrenia. SNPs, single nucleotide polymorphisms; OR, odds ratio; CI, confidence interval.

In the reverse MR analysis, results from the IVW method revealed that no form of dementia was found to be a risk factor for schizophrenia. (ACD-schizophrenia, OR = 1.006, 95% CI: 0.976 ～ 1.038, *P* = 0.691, FDR-corrected *P* = 0.691; AD-schizophrenia, OR = 1.009, 95% CI: 0.979 ～ 1.040, *P* = 0.546, FDR-corrected *P* = 0.682; VaD-schizophrenia, OR = 1.017, 95% CI: 0.990 ～ 1.045, *P* = 0.217, FDR-corrected *P* = 0.362; FTD-schizophrenia, OR = 0.986, 95% CI: 0.968 ～ 1.005, *P* = 0.140, FDR-corrected *P* = 0.350; DLB-schizophrenia, OR = 1.020, 95% CI: 0.999 ～ 1.041, *P* = 0.059, FDR-corrected *P* = 0.295) (Fig 3).

## Discussion

In this MR study, we assessed the causal relationship between schizophrenia and five types of dementia. Results from the IVW suggested that schizophrenia is associated with an increased risk of ACD, AD, and VaD. And no form of dementia assessed in this study was found to be a risk factor for schizophrenia. These results suggest that early prevention of schizophrenia may be a potential approach to prevent ACD, AD, and VaD.

Our findings suggest that schizophrenia may be a risk factor for ACD, AD, and VaD, aligning with previous observational studies. So far, the exact mechanism by which schizophrenia affects dementia remains unclear. Scholars have proposed several hypotheses to explain the association between schizophrenia and increased dementia risk. One hypothesis is that structural brain abnormalities and aging in patients living with schizophrenia may contribute to premature onset of dementia [41,42]. A study found that strikingly similar microstructural deficits in the white matter of patients living with schizophrenia and AD [43]. This finding provides evidence of brain structural changes leading to cognitive deficits in schizophrenia, which are similar to those observed in dementia. Additionally, schizophrenia is sometimes hypothesized to be a disease of accelerated aging, which may explain the high incidence of dementia in patients living with schizophrenia. A study using neuroimaging has shown patients living with schizophrenia age their brains more than their chronological age [44]. A large meta-analysis from the ENIGMA consortium found that patients living with schizophrenia had structural brain measurements equivalent to those of individuals more than three and a half years older than healthy controls [45]. Second, metabolic dysfunction might contribute to premature dementia in patients living with schizophrenia. A study indicated that over 50% of individuals with psychiatric disorders are affected by obesity, 39% have hypertension, and 19–39% suffer from dyslipidemia [46]. These risk factors can lead to atherosclerosis, which, by narrowing cerebral arteries, increases the risk of ischemia and stroke, potentially leading to VaD. Third, psychotropic medications may exacerbate cognitive impairment in patients living with schizophrenia. Prolonged exposure to antipsychotic drugs can contribute to early dementia [47]. Antipsychotic drugs may affect dementia by altering and disrupting cortical dopaminergic circuits, which are also implicated in cognitive decline in dementia [48]. These drugs reduce dopaminergic activity of D2 receptors, thereby decreasing D2 signaling in the striatum and reducing neuron survival in this circuit. Additionally, antipsychotics may increase the risk of dementia through their anticholinergic effects, as anticholinergic drugs have been shown to elevate dementia risk in the general population and are linked to cognitive impairment in patients living with schizophrenia [49].

No causal association was found between schizophrenia and FTD or DLB, according to this MR study. Nevertheless, observational studies pointed to an association between schizophrenia and FTD or DLB. We believe that this contradiction may be related to two reasons. Firstly, these contradictions may be related to the inherent limits of observational studies, such as various confounders, biases, and reverse causality, which can all lead to inaccurate results in observational studies [50]. Secondly, these contradictions may also be related to the small number of cases in the GWAS database we used, despite using the largest and latest GWAS database, in comparison to population-based observational studies, the sample size of this database was relatively small. In the future, studies with more extensive sample sizes may be necessary to further investigate the causal association between schizophrenia and FTD and DLB.

Furthermore, our findings also suggest that no form of dementia was a risk factor for schizophrenia. In the first case report describing dementia, Alois Alzheimer noted that patients living with dementia exhibited psychiatric symptoms, including paranoid delusions and hallucinations [51]. Zubenko et al. found that psychiatric symptoms in patients living with AD were associated with increased cortical neurodegeneration, elevated subcortical norepinephrine levels, and reduced cortical and subcortical serotonin/5-HIAA levels [52], suggesting a neurochemical and neuropathological link between AD and psychosis. Emerging evidence suggests that degeneration of monoaminergic neurons, and subsequent compensatory changes, may underlie the neuropsychiatric symptoms observed in AD [53]. Notwithstanding these findings, there is still a lack of robust clinical evidence demonstrating a causal relationship between schizophrenia and AD. Furthermore, research on the association between schizophrenia and other types of dementia (e.g., ACD, VaD, FTD, DLB) remains limited, with insufficient evidence to establish a causal relationship. This warrants further exploration in future studies.

Our MR study possesses numerous strengths. First, in comparison to previous observational studies, MR study reduces influence of reverse causality and confounding factors. Second, we applied the Benjamini Hochberg technique for FDR correction in order to lower the possibility of false positives. Finally, although previous MR studies have explored the potential causal relationship between schizophrenia and AD, they have certain limitations in terms of data timeliness, the stringency of IVs selection, and the coverage of dementia subtypes. For example, Wei et al. conducted a bidirectional MR analysis using the 2013 schizophrenia GWAS data (N = 77,096) and the 2019 AD GWAS data (N = 63,926) [16], and found no causal relationship between the two. Liu et al. conducted an MR analysis using the 2022 schizophrenia GWAS data (N = 130,644) and the 2019 AD GWAS data (N = 63,926) [18], and still found no causal relationship between the two. In addition, Ancha Baranova et al. conducted an MR analysis using the 2022 schizophrenia (N = 130,644) GWAS data and the 2022 AD (N = 487,511) GWAS data [17], and selected IVs under the conditions of r^2^ < 0.01 within a clumping distance of 10,000 kb. The results indicated that schizophrenia is associated with an increased risk of AD. Building on previous work, our MR study used the same data as Ancha Baranova et al. (schizophrenia N = 130,644, AD N = 487,511), and applied more stringent selection criteria for IVs (r^2^ < 0.001 within a clumping distance of 10,000 kb). We also included an evaluation of the causal relationship between schizophrenia and multiple dementia subtypes (ACD, VaD, FTD, DLB). Our results showed that schizophrenia is associated with an increased risk of ACD, AD, and VaD, providing multidimensional new insights into the potential genetic relationship between schizophrenia and dementia.

It is important to note that our study is susceptible to the following limitations: Firstly, due to the fact that our study relied on GWAS data from individuals belonging to the European population, it limits the applicability of our findings to other populations. Therefore, caution is needed when applying these results to different populations. Secondly, the study lacked formal mediation analyses to explore potential pathways between schizophrenia and dementia. Thirdly, we cannot completely eliminate the influence of outliers, although we have instituted rigorous measures to identify outliers and reduce horizontal pleiotropy. This might be ascribed to the complicated and ambiguous biological functions of many variations in genetics. Fourth, although we utilized the largest and most recent GWAS data, the sample sizes for FTD and DLB cases remain relatively small. Future studies with larger sample sizes may be needed to further investigate the causal relationships between schizophrenia and DLB or FTD. Fifth, from a clinical and research perspective, patients living with schizophrenia are generally classified into early-onset and late-onset types. Both types share common characteristics, such as cognitive deficits and psychotic symptoms. However, patients living with late-onset schizophrenia are more likely to be married, possess a superior career history, and present with a paranoid subtype in comparison to those with early-onset schizophrenia. Patients living with late-onset schizophrenia are more likely to experience dementia in the future [54]. A comprehensive comparison of the causal relationship between two types of schizophrenia and dementia could help us develop more targeted treatment strategies. However, the lack of detailed information on disease type limited our ability to conduct further analyses, which could be explored in future studies.

## Conclusion

In conclusion, our findings indicate that schizophrenia is associated with an increased risk of ACD, AD, and VaD. Our study enhances the genetic understanding of the causal relationship between schizophrenia and dementia, addressing a gap in the existing academic literature. Future studies are needed to further explore the shared biological mechanisms between schizophrenia and dementia and to utilize higher-quality GWAS data to validate and refine these findings.

## Supporting information

S1 File STROBE-MR-checklist. (DOCX)

S2 File The scatter plot of Mendelian randomization analysis results. (DOCX)

S3 File The funnel plot of Mendelian randomization analysis results. (DOCX)

S4 File The leave-one-out analyses of Mendelian randomization analysis results. (DOCX)

S1 Table SNP deleted from Mendelian randomization analysis. (XLSX)

S2 Table SNPs used in Mendelian randomization analysis. (XLSX)
