# Supplementary material for: Causal relationship between schizophrenia and five types of dementia: A bidirectional two-sample Mendelian randomization study
Source: PLoS One. 2025 May 8;20(5):e0322752. doi: 10.1371/journal.pone.0322752 (PMC12061177; doi:10.1371/journal.pone.0322752)
Supplement: S1 File — (DOCX) [file pone.0322752.s001.docx]

**STROBE-MR checklist of recommended items to address in reports of Mendelian randomization studies**^1^ ^2^

| **Item No.** | **Section** | **Checklist item** | **Page No.** | **Relevant text from manuscript** |
| --- | --- | --- | --- | --- |
| 1 | **TITLE and ABSTRACT** | Indicate Mendelian randomization (MR) as the study’s design in the title and/or the abstract if that is a main purpose of the study | 1-2 | Causal relationship between schizophrenia and five types of dementia: A bidirectional two-sample Mendelian randomization study  Background: Although observational research indicates an association between schizophrenia and dementia, it is unclear whether the two are causally related. In order to examine the causal relationship between schizophrenia and five types of dementia (all-cause dementia, Alzheimer's disease, vascular dementia, frontotemporal dementia, and dementia with Lewy bodies), we performed a bidirectional two-sample Mendelian randomization analysis.  Methods: In this study, pooled statistics of schizophrenia and dementia were obtained from the Large-scale genome-wide association study (GWAS) in individuals of European ancestry. Inverse variance weighted (IVW) was the primary statistical method used in this Mendelian randomization. To further support our findings, we also used MR-Egger, weighted median, and cML-MA. We also used a number of sensitivity analyses to evaluate pleiotropy and heterogeneity.  Results: In the study of the effect of schizophrenia on dementia, findings from the IVW analysis suggested that schizophrenia is associated with an increased risk of all-cause dementia (OR=1.065, 95%CI: 1.027～1.104, P=0.001, FDR-corrected P=0.003), Alzheimer's disease (OR=1.029, 95%CI: 1.003～1.054，P=0.027, FDR-corrected P=0.045), and vascular dementia (OR=1.106, 95%CI: 1.023～1.197，P=0.012, FDR-corrected P=0.029). In the study of the effect of dementia on schizophrenia, no form of dementia assessed in this study was found to be a risk factor for schizophrenia.  Conclusion: Our findings suggest that schizophrenia may be a risk factor for all-cause dementia, Alzheimer's disease, and vascular dementia, but no dementia of any kind was found to be a risk factor for schizophrenia. Our study provides insights into the potential genetic relationship between schizophrenia and dementia. |
|  | **INTRODUCTION** |  |  |  |
| 2 | **Background** | Explain the scientific background and rationale for the reported study. What is the exposure? Is a potential causal relationship between exposure and outcome plausible? Justify why MR is a helpful method to address the study question | 2-5 | Dementia is a common neurodegenerative disease characterized by the progressive decline of cognitive and memory functions, encompassing conditions such as all-cause dementia (ACD), Alzheimer's disease (AD), vascular dementia (VaD), frontotemporal dementia (FTD), and dementia with Lewy bodies (DLB) [1]. With the global aging population, the incidence of dementia is rising annually. Recent projections indicate that by 2050, the number of patients living with dementia worldwide will escalate from 574,000 cases in 2019 to 1,528,000 cases [2, 3]. Meanwhile, expenditures due to dementia will reach 1.6 trillion dollars [4] which will result in a huge financial, social, and public health burden. It has been found that in addition to cognitive decline, patients living with dementia experience psychological symptoms such as psychosis, agitation, anxiety and depression [5]. However, it is unclear whether dementia contributes to the onset of schizophrenia.  Schizophrenia, a chronic psychiatric disorder with symptoms of hallucinations and delusions, is one of the most disabling and economically costly disorders worldwide, affecting about 1% of the population [6]. The disease is linked to deficits in perception, communication, cognitive processes, and deviations in behavior [7, 8], which often result in social disability and considerable dysfunction. Multiple investigations have reported a notable cognitive deterioration over time in patients living with schizophrenia [9, 10] and cognitive impairment is also regarded as a core feature of the disorder. Many studies have indicated an association between schizophrenia and dementia. For example, a meta-analysis of cohort studies indicated that patients living with schizophrenia have a higher risk of developing dementia compared to those without schizophrenia (relative risk [RR] = 2.29; 95% confidence interval [CI]: 1.35~3.88) [11]. A cohort study in the United States that included 8,011,773 participants found that at the age of 66, the prevalence of dementia in the schizophrenia group was 21.5 times higher than that in the non-schizophrenia group, and at the age of 80, it was 6.2 times higher [12]. In addition, a large German case-control study that included 1,686,759 patients living with dementia confirmed multiple risk factors for dementia and noted that schizophrenia is associated with an increased risk of dementia [13].  However, the conclusions of observational studies are easily influenced by confounding factors and reverse causality, making them insufficient to provide support for causality. Furthermore, meticulously planned randomized controlled trials (RCTs) are typically considered to be the most reliable method for causal inference, but they frequently encounter constraints in terms of costs and time. Mendelian randomization (MR) is a powerful epidemiological method that treats single nucleotide polymorphisms (SNPs) as instrumental variables (IVs) to infer causality [14]. Because genetic variations are randomly arranged at conception, MR reduces the influence of confounding factors and reverse causality, achieving the same effect as RCTs and substantially lowering research costs [15]. The Genome-Wide Association Study (GWAS) database is an extensively utilized open database that supplies data regarding diseases and their genetic correlations. In the past, three MR studies have explored the causal relationship between multiple mental disorders and AD [16-18]. However, due to the comprehensive updates to the GWAS of schizophrenia and AD, and the lack of studies exploring the causal relationship between schizophrenia and other subtypes of dementia, further investigations of the causal relationship between schizophrenia and other subtypes of dementia are needed. In this study, we used a bidirectional two-sample MR study to examine the causal relationship between schizophrenia and five dementias, leveraging aggregated statistical data from the GWAS dataset. |
| 3 | **Objectives** | State specific objectives clearly, including pre-specified causal hypotheses (if any). State that MR is a method that, under specific assumptions, intends to estimate causal effects | 5 | In this study, we used a bidirectional two-sample MR study to examine the causal relationship between schizophrenia and five dementias, leveraging aggregated statistical data from the GWAS dataset. |
|  | **METHODS** |  |  |  |
| 4 | **Study design and data sources** | Present key elements of the study design early in the article. Consider including a table listing sources of data for all phases of the study. For each data source contributing to the analysis, describe the following: |  |  |
|  | a) | Setting: Describe the study design and the underlying population, if possible. Describe the setting, locations, and relevant dates, including periods of recruitment, exposure, follow-up, and data collection, when available. | 5-6 | The schematic diagram of the MR analysis is shown in Fig 1.  The MR study must adhere to the following assumptions: 1) the assumption of relevance: IVs should be strongly associated with exposure; 2) the assumption of independence: IVs should not be associated with any confounding factors; 3) the assumption of exclusivity: IVs can only affect outcomes through exposure and no other pathways.  The GWAS pooled data for schizophrenia was obtained from the Psychiatric Genomics Consortium (PGC), which includes 53,386 cases of schizophrenia and 77,258 controls [19]. The GWAS dataset for ACD was collected from the FinnGen Consortium, which includes 18,216 cases of ACD and 77,258 controls. The GWAS dataset for AD was derived from stage I of the European Alzheimer’s and Dementia Biobank (EADB), including 85,934 cases (39,106 clinically diagnosed cases and 46,828 proxy cases) and 401,577 controls [21]. The GWAS dataset for VaD was also collected from the FinnGen Consortium, which includes 3,116 cases of VaD and 433,066 controls. The GWAS dataset for FTD was derived from research comprising 3024 individuals (515 cases of FTD and 2509 controls) [22]. The GWAS summary statistics for DLB were acquired from a study done by Ruth Chia et al. This study comprised 6,618 individuals, consisting of 2,591 cases of DLB and 4,027 controls [23]. |
|  | b) | Participants: Give the eligibility criteria, and the sources and methods of selection of participants. Report the sample size, and whether any power or sample size calculations were carried out prior to the main analysis | 6 | The GWAS pooled data for schizophrenia was obtained from the Psychiatric Genomics Consortium (PGC), which includes 53,386 cases of schizophrenia and 77,258 controls [19]. The GWAS dataset for ACD was collected from the FinnGen Consortium, which includes 18,216 cases of ACD and 77,258 controls. The GWAS dataset for AD was derived from stage I of the European Alzheimer’s and Dementia Biobank (EADB), including 85,934 cases (39,106 clinically diagnosed cases and 46,828 proxy cases) and 401,577 controls [21]. The GWAS dataset for VaD was also collected from the FinnGen Consortium, which includes 3,116 cases of VaD and 433,066 controls. The GWAS dataset for FTD was derived from research comprising 3024 individuals (515 cases of FTD and 2509 controls) [22]. The GWAS summary statistics for DLB were acquired from a study done by Ruth Chia et al. This study comprised 6,618 individuals, consisting of 2,591 cases of DLB and 4,027 controls [23]. |
|  | c) | Describe measurement, quality control and selection of genetic variants | 7-8 | To fulfil the three key assumptions, we have developed a strict protocol for selecting IVs. First, for the schizophrenia-dementia study, we set the significance threshold at p < 5 × 10−8. However, for the dementia-schizophrenia study, only a few SNPs met this stringent threshold (p < 5 × 10−8), so we used a lower standard (p < 5 × 10−6). In the past MR studies, such thresholds are commonly used when SNPs counts are limited [24, 25]. Second, we used linkage disequilibrium (LD) clustering to identify independent SNPs meeting the specified criteria (LD R2 < 0.001, LD distance = 10,000 kb). Third, to minimize the influence of weak instrument bias, we removed SNPs having F-values below 10. The F-value was computed using the following formula:  F=R^2 ((N-2))/((1-R^2 ) )  Where N is the sample size and R2 represents the exposure variance explained by genetic variation. The specific formula used to calculate R2 is as follows:  R^2=2×EAF×(1-EAF)×β^2  Where β represents the effect estimate of the genetic variance in the exposed GWAS, and EAF is the effect allele frequency [26]. Fourth, we excluded IVs that showed a strong association with the outcome (p < 5 × 10⁻⁵). Fifth, we harmonized and integrated the selected datasets of exposure and outcome to guarantee that the effect alleles belonged to the same allele, and eliminated palindromic SNPs. Sixth, we conducted a Steiger test to filter out reverse causal SNPs, excluding those that tested in the “FALSE” direction [27]. Finally, we queried the phenotype information associated with SNPs using the NHGRI-EBI Catalog (https://www.ebi.ac.uk/gwas/) and removed SNPs associated with potential confounders. |
|  | d) | For each exposure, outcome, and other relevant variables, describe methods of assessment and diagnostic criteria for diseases | N/A |  |
|  | e) | Provide details of ethics committee approval and participant informed consent, if relevant | 5 | The original GWAS already had patient informed consent and ethical approvals. Therefore, no additional ethical approval was needed for this study. |
| 5 | **Assumptions** | Explicitly state the three core IV assumptions for the main analysis (relevance, independence and exclusion restriction) as well assumptions for any additional or sensitivity analysis | 5 | The MR study must adhere to the following assumptions: 1) the assumption of relevance: IVs should be strongly associated with exposure; 2) the assumption of independence: IVs should not be associated with any confounding factors; 3) the assumption of exclusivity: IVs can only affect outcomes through exposure and no other pathways. |
| 6 | **Statistical methods: main analysis** | Describe statistical methods and statistics used |  |  |
|  | a) | Describe how quantitative variables were handled in the analyses (i.e., scale, units, model) | 7-8 | To fulfil the three key assumptions, we have developed a strict protocol for selecting IVs. First, for the schizophrenia-dementia study, we set the significance threshold at p < 5 × 10−8. However, for the dementia-schizophrenia study, only a few SNPs met this stringent threshold (p < 5 × 10−8), so we used a lower standard (p < 5 × 10−6). In the past MR studies, such thresholds are commonly used when SNPs counts are limited [24, 25]. Second, we used linkage disequilibrium (LD) clustering to identify independent SNPs meeting the specified criteria (LD R2 < 0.001, LD distance = 10,000 kb). Third, to minimize the influence of weak instrument bias, we removed SNPs having F-values below 10. The F-value was computed using the following formula:  F=R^2 ((N-2))/((1-R^2 ) )  Where N is the sample size and R2 represents the exposure variance explained by genetic variation. The specific formula used to calculate R2 is as follows:  R^2=2×EAF×(1-EAF)×β^2  Where β represents the effect estimate of the genetic variance in the exposed GWAS, and EAF is the effect allele frequency [26]. Fourth, we excluded IVs that showed a strong association with the outcome (p < 5 × 10⁻⁵). Fifth, we harmonized and integrated the selected datasets of exposure and outcome to guarantee that the effect alleles belonged to the same allele, and eliminated palindromic SNPs. Sixth, we conducted a Steiger test to filter out reverse causal SNPs, excluding those that tested in the “FALSE” direction [27]. Finally, we queried the phenotype information associated with SNPs using the NHGRI-EBI Catalog (https://www.ebi.ac.uk/gwas/) and removed SNPs associated with potential confounders. |
|  | b) | Describe how genetic variants were handled in the analyses and, if applicable, how their weights were selected | 7-8 | To fulfil the three key assumptions, we have developed a strict protocol for selecting IVs. First, for the schizophrenia-dementia study, we set the significance threshold at p < 5 × 10−8. However, for the dementia-schizophrenia study, only a few SNPs met this stringent threshold (p < 5 × 10−8), so we used a lower standard (p < 5 × 10−6). In the past MR studies, such thresholds are commonly used when SNPs counts are limited [24, 25]. Second, we used linkage disequilibrium (LD) clustering to identify independent SNPs meeting the specified criteria (LD R2 < 0.001, LD distance = 10,000 kb). Third, to minimize the influence of weak instrument bias, we removed SNPs having F-values below 10. The F-value was computed using the following formula:  F=R^2 ((N-2))/((1-R^2 ) )  Where N is the sample size and R2 represents the exposure variance explained by genetic variation. The specific formula used to calculate R2 is as follows:  R^2=2×EAF×(1-EAF)×β^2  Where β represents the effect estimate of the genetic variance in the exposed GWAS, and EAF is the effect allele frequency [26]. Fourth, we excluded IVs that showed a strong association with the outcome (p < 5 × 10⁻⁵). Fifth, we harmonized and integrated the selected datasets of exposure and outcome to guarantee that the effect alleles belonged to the same allele, and eliminated palindromic SNPs. Sixth, we conducted a Steiger test to filter out reverse causal SNPs, excluding those that tested in the “FALSE” direction [27]. Finally, we queried the phenotype information associated with SNPs using the NHGRI-EBI Catalog (https://www.ebi.ac.uk/gwas/) and removed SNPs associated with potential confounders. |
|  | c) | Describe the MR estimator (e.g. two-stage least squares, Wald ratio) and related statistics. Detail the included covariates and, in case of two-sample MR, whether the same covariate set was used for adjustment in the two samples | 8-9 | This MR study utilized four statistical methods: Inverse variance weighted (IVW), MR Egger, Weighted median (WM) and cML-MA.  Of these, the IVW is the primary statistical method for our MR study, which assumes that IVs affect outcomes exclusively through specific exposures, employing the meta-analysis approach to integrate Wald ratios of individual SNPs. In the absence of horizontal pleiotropy and heterogeneity, IVW linear regression provides unbiased causal estimates [28]. The WM is expected to offer reliable causal estimates even if up to 50% of the IVs are invalid [29]. The MR-Egger can assess whether genetic variants have pleiotropic effects on the outcome and provide a consistent estimate of the causal effect [30]. However, the MR-Egger method often has bias and low efficacy, even if its appropriateness for MR in the presence of horizontal pleiotropy. The cML-MA technique is a novel methodology. We employed the cML-MA approach to tackle correlated and uncorrelated pleiotropic effects, and it was believed to be more potent than MR-Egger, with superior type I error control, and could reach greater statistical efficacy [31]. |
|  | d) | Explain how missing data were addressed | N/A |  |
|  | e) | If applicable, indicate how multiple testing was addressed | N/A |  |
| 7 | **Assessment of assumptions** | Describe any methods or prior knowledge used to assess the assumptions or justify their validity | 9 | We used several methods for sensitivity analysis. Both the IVW and MR-Egger were utilized in the execution of the Cochran's Q test [28], which was designed to assess the heterogeneity in causality (p < 0.05 indicates presence of heterogeneity). Then, the MR-Egger intercept [30] and MR-Pleiotropy Residual Sum and Outlier method (MR-PRESSO) Global Test [32] were employed to assess pleiotropy (p<0.05 indicates presence of pleiotropy). If there is heterogeneity or pleiotropy, we detect potential outliers using the MR-PRESSO [32] and Radial MR [33] methods. If potential outliers were found, we discarded them and re-executed the MR analysis. The Leave-one-out analysis was also carried out in our MR study, this technique consisted of eliminating one SNP at a moment; if influential SNPs were present, we treated the results with caution; otherwise, the results were considered robust.  We applied the Benjamini Hochberg technique for FDR correction in order to lower the possibility of false positives [34]. There is a significant causal relationship when the FDR-corrected P < 0.05 and the original P < 0.05 [35]. RStudio was utilized to conduct MR analysis, with TwoSample MR (version 0.5.8) being the R package utilized. |
| 8 | **Sensitivity analyses and additional analyses** | Describe any sensitivity analyses or additional analyses performed (e.g. comparison of effect estimates from different approaches, independent replication, bias analytic techniques, validation of instruments, simulations) | 9 | We used several methods for sensitivity analysis. Both the IVW and MR-Egger were utilized in the execution of the Cochran's Q test [28], which was designed to assess the heterogeneity in causality (p < 0.05 indicates presence of heterogeneity). Then, the MR-Egger intercept [30] and MR-Pleiotropy Residual Sum and Outlier method (MR-PRESSO) Global Test [32] were employed to assess pleiotropy (p<0.05 indicates presence of pleiotropy). If there is heterogeneity or pleiotropy, we detect potential outliers using the MR-PRESSO [32] and Radial MR [33] methods. If potential outliers were found, we discarded them and re-executed the MR analysis. The Leave-one-out analysis was also carried out in our MR study, this technique consisted of eliminating one SNP at a moment; if influential SNPs were present, we treated the results with caution; otherwise, the results were considered robust.  We applied the Benjamini Hochberg technique for FDR correction in order to lower the possibility of false positives [34]. There is a significant causal relationship when the FDR-corrected P < 0.05 and the original P < 0.05 [35]. RStudio was utilized to conduct MR analysis, with TwoSample MR (version 0.5.8) being the R package utilized. |
| 9 | **Software and pre-registration** |  |  |  |
|  | a) | Name statistical software and package(s), including version and settings used | 9 | RStudio was utilized to conduct MR analysis, with TwoSample MR (version 0.5.8) being the R package utilized. |
|  | b) | State whether the study protocol and details were pre-registered (as well as when and where) | N/A |  |
|  | **RESULTS** |  |  |  |
| 10 | **Descriptive data** |  |  |  |
|  | a) | Report the numbers of individuals at each stage of included studies and reasons for exclusion. Consider use of a flow diagram | 10-12,  Fig 1 | In the schizophrenia-dementia study, we excluded SNPs with LD, removed SNPs strongly associated with the outcome or were palindromic (S1 Table), and retained SNPs with F-value > 10. We then conducted a Steiger test to exclude SNPs likely to exhibit reverse causality (S1 Table). Then we removed SNPs related to potential confounding factors (smoking, drinking, hypertension, diabetes, body mass index [36-40]) (S1 Table). Finally, we employed MR-PRESSO and Radial MR methods to identify and exclude any potential outliers (S1 Table). The remaining SNPs were then used in MR analysis, with detailed information provided in S2 Table.  In the dementia-schizophrenia study, we applied the same methods to select IVs and found no SNP associated with confounders for schizophrenia. The ultimate SNPs employed in the MR analysis are enumerated in S2 Table. |
|  | b) | Report summary statistics for phenotypic exposure(s), outcome(s), and other relevant variables (e.g. means, SDs, proportions) | N/A |  |
|  | c) | If the data sources include meta-analyses of previous studies, provide the assessments of heterogeneity across these studies | N/A |  |
|  | d) | For two-sample MR:  i.  Provide justification of the similarity of the genetic variant-exposure associations between the exposure and outcome samples  ii.  Provide information on the number of individuals who overlap between the exposure and outcome studies | NA |  |
| 11 | **Main results** |  |  |  |
|  | a) | Report the associations between genetic variant and exposure, and between genetic variant and outcome, preferably on an interpretable scale | N/A |  |
|  | b) | Report MR estimates of the relationship between exposure and outcome, and the measures of uncertainty from the MR analysis, on an interpretable scale, such as odds ratio or relative risk per SD difference | 10-12 | Results from the IVW method revealed that schizophrenia is associated with an increased risk of ACD (OR=1.065, 95%CI: 1.027～1.104, P=0.001, FDR-corrected P=0.003), AD (OR=1.029, 95%CI: 1.003～1.054, P=0.027, FDR-corrected P=0.045), and VaD (OR=1.106, 95%CI: 1.023～1.197, P=0.012, FDR-corrected P=0.029). In contrast, no causal association was found between schizophrenia and FTD (OR=0.717, 95%CI: 0.343～1.500, P=0.377, FDR-corrected P=0.471) or DLB (OR=0.966, 95%CI: 0.858～1.087, P=0.566, FDR-corrected P=0.566) (Fig 2).  I In the reverse MR analysis, results from the IVW method revealed that no form of dementia was found to be a risk factor for schizophrenia (ACD-schizophrenia, OR=1.006, 95% CI: 0.976～1.038, P=0.691, FDR-corrected P=0.691; AD-schizophrenia, OR=1.009, 95% CI: 0.979～1.040, P=0.546, FDR-corrected P=0.682; VaD-schizophrenia, OR=1.017, 95% CI: 0.990～1.045, P=0.217, FDR-corrected P=0.362; FTD-schizophrenia, OR=0.986, 95% CI: 0.968～1.005, P=0.140, FDR-corrected P=0.350; DLB-schizophrenia, OR=1.020, 95% CI: 0.999～1.041, P=0.059, FDR-corrected P=0.295) (Fig 3). |
|  | c) | If relevant, consider translating estimates of relative risk into absolute risk for a meaningful time period | N/A |  |
|  | d) | Consider plots to visualize results (e.g. forest plot, scatterplot of associations between genetic variants and outcome versus between genetic variants and exposure) | - |  |
| 12 | **Assessment of assumptions** |  |  |  |
|  | a) | Report the assessment of the validity of the assumptions | 5 | The MR study must adhere to the following assumptions: 1) the assumption of relevance: IVs should be strongly associated with exposure; 2) the assumption of independence: IVs should not be associated with any confounding factors; 3) the assumption of exclusivity: IVs can only affect outcomes through exposure and no other pathways. |
|  | b) | Report any additional statistics (e.g., assessments of heterogeneity across genetic variants, such as *I^2^*, Q statistic or E-value) | 11 | The associations between schizophrenia and ACD, AD, and VaD did not show any signs of heterogeneity (P > 0.05) or pleiotropy (P > 0.05), according to sensitivity analysis (Table 2, S2 File). The funnel plot displayed a symmetrical distribution of SNPs (S3 File). The leave-one-out analysis displayed consistent results upon the exclusion of individual SNPs, with no single SNP exerting disproportionate influence on the overall estimate, thereby demonstrating the results robustness (S4 File). |
| 13 | **Sensitivity analyses and additional analyses** |  |  |  |
|  | a) | Report any sensitivity analyses to assess the robustness of the main results to violations of the assumptions | 11, Table 2 | The associations between schizophrenia and ACD, AD, and VaD did not show any signs of heterogeneity (P > 0.05) or pleiotropy (P > 0.05), according to sensitivity analysis (Table 2, S2 File). The funnel plot displayed a symmetrical distribution of SNPs (S3 File). The leave-one-out analysis displayed consistent results upon the exclusion of individual SNPs, with no single SNP exerting disproportionate influence on the overall estimate, thereby demonstrating the results robustness (S4 File). |
|  | b) | Report results from other sensitivity analyses or additional analyses | 11, S2 File, S3 File, S4 File | The leave-one-out analysis displayed consistent results upon the exclusion of individual SNPs, with no single SNP exerting disproportionate influence on the overall estimate, thereby demonstrating the results robustness (S4 File). |
|  | c) | Report any assessment of direction of causal relationship (e.g., bidirectional MR) | 11-12, Fig 3 | In the reverse MR analysis, results from the IVW method revealed that no form of dementia was found to be a risk factor for schizophrenia (ACD-schizophrenia, OR=1.006, 95% CI: 0.976～1.038, P=0.691, FDR-corrected P=0.691; AD-schizophrenia, OR=1.009, 95% CI: 0.979～1.040, P=0.546, FDR-corrected P=0.682; VaD-schizophrenia, OR=1.017, 95% CI: 0.990～1.045, P=0.217, FDR-corrected P=0.362; FTD-schizophrenia, OR=0.986, 95% CI: 0.968～1.005, P=0.140, FDR-corrected P=0.350; DLB-schizophrenia, OR=1.020, 95% CI: 0.999～1.041, P=0.059, FDR-corrected P=0.295) (Fig 3). |
|  | d) | When relevant, report and compare with estimates from non-MR analyses | N/A |  |
|  | e) | Consider additional plots to visualize results (e.g., leave-one-out analyses) | 10, S4 File | The leave-one-out analysis displayed consistent results upon the exclusion of individual SNPs, with no single SNP exerting disproportionate influence on the overall estimate, thereby demonstrating the results robustness (S4 File). |
|  | **DISCUSSION** |  |  |  |
| 14 | **Key results** | Summarize key results with reference to study objectives | 10-11 | In this MR study, we assessed the causal relationship between schizophrenia and five types of dementia. Results from the IVW suggested that schizophrenia is associated with an increased risk of ACD, AD, and VaD. And no form of dementia assessed in this study was found to be a risk factor for schizophrenia. These results suggest that early prevention of schizophrenia may be a potential approach to prevent ACD, AD, and VaD. |
| 15 | **Limitations** | Discuss limitations of the study, taking into account the validity of the IV assumptions, other sources of potential bias, and imprecision. Discuss both direction and magnitude of any potential bias and any efforts to address them | 16-17 | It is important to note that our study is susceptible to the following limitations: Firstly, due to the fact that our research relied on GWAS data from individuals belonging to the European population, it limits the applicability of our findings to other populations. Therefore, caution is needed when applying these results to different populations. Secondly, the study lacked formal mediation analyses to explore potential pathways between schizophrenia and dementia. Thirdly, we cannot completely eliminate the influence of outliers, although we have instituted rigorous measures to identify outliers and reduce horizontal pleiotropy. This might be ascribed to the complicated and ambiguous biological functions of many variations in genetics. Fourth, from a clinical and research perspective, patients living with schizophrenia are generally classified into early-onset and late-onset types. Both types share common characteristics, such as cognitive deficits and psychotic symptoms. However, patients living with late-onset schizophrenia are more likely to be married, possess a superior career history, and present with a paranoid subtype in comparison to those with early-onset schizophrenia. Patients living with late-onset schizophrenia are more likely to experience dementia in the future [58]. A comprehensive comparison of the causal relationship between two types of schizophrenia and dementia could help us develop more targeted treatment strategies. However, the lack of detailed information on disease type limited our ability to conduct further analyses, which could be explored in future studies. |
| 16 | **Interpretation** |  |  |  |
|  | a) | Meaning: Give a cautious overall interpretation of results in the context of their limitations and in comparison with other studies | 12-15 | Our findings suggest that schizophrenia may be a risk factor for ACD, AD, and VaD, aligning with previous observational studies. So far, the exact mechanism by which schizophrenia affects dementia remains unclear. Scholars have proposed several hypotheses to explain the association between schizophrenia and increased dementia risk. One hypothesis is that structural brain abnormalities and aging in patients living with schizophrenia may contribute to premature onset of dementia [41, 42]. A study found that strikingly similar microstructural deficits in the white matter of patients living with schizophrenia and AD [43]. This finding provides evidence of brain structural changes leading to cognitive deficits in schizophrenia, which are similar to those observed in dementia. Additionally, schizophrenia is sometimes hypothesized to be a disease of accelerated aging, which may explain the high incidence of dementia in patients living with schizophrenia. A study using neuroimaging has shown patients living with schizophrenia age their brains more than their chronological age [44]. A large meta-analysis from the ENIGMA consortium found that patients living with schizophrenia had structural brain measurements equivalent to those of individuals more than three and a half years older than healthy controls [45]. Second, metabolic dysfunction might contribute to premature dementia in patients living with schizophrenia. A study indicated that over 50% of individuals with psychiatric disorders are affected by obesity, 39% have hypertension, and 19-39% suffer from dyslipidemia [46]. These risk factors can lead to atherosclerosis, which, by narrowing cerebral arteries, increases the risk of ischemia and stroke, potentially leading to VaD. Third, psychotropic medications may exacerbate cognitive impairment in patients living with schizophrenia. Prolonged exposure to antipsychotic drugs can contribute to early dementia [47]. Antipsychotic drugs may affect dementia by altering and disrupting cortical dopaminergic circuits, which are also implicated in cognitive decline in dementia [48]. These drugs reduce dopaminergic activity of D2 receptors, thereby decreasing D2 signaling in the striatum and reducing neuron survival in this circuit. Additionally, antipsychotics may increase the risk of dementia through their anticholinergic effects, as anticholinergic drugs have been shown to elevate dementia risk in the general population and are linked to cognitive impairment in patients living with schizophrenia [49].  No causal association was found between schizophrenia and FTD or DLB, according to this MR study. Nevertheless, observational studies pointed to an association between schizophrenia and FTD or DLB. We believe that this contradiction may be related to two reasons. Firstly, these contradictions may be related to the inherent limits of observational studies, such as various confounders, biases, and reverse causality, which can all lead to inaccurate results in observational studies [50]. Secondly, these contradictions may also be related to the small number of cases in the GWAS database we used, despite using the largest and latest GWAS database, in comparison to population-based observational studies, the sample size of this database was relatively small. In the future, studies with more extensive sample sizes may be necessary to further investigate the causal association between schizophrenia and FTD and DLB.  Furthermore, our findings also suggest that no form of dementia was a risk factor for schizophrenia. In the first case report describing dementia, Alois Alzheimer noted that patients living with dementia exhibited psychiatric symptoms, including paranoid delusions and hallucinations [51]. Zubenko et al. found that psychiatric symptoms in patients living with AD were associated with increased cortical neurodegeneration, elevated subcortical norepinephrine levels, and reduced cortical and subcortical serotonin/5-HIAA levels [52], suggesting a neurochemical and neuropathological link between AD and psychosis. Emerging evidence suggests that degeneration of monoaminergic neurons, and subsequent compensatory changes, may underlie the neuropsychiatric symptoms observed in AD [53]. Notwithstanding these findings, there is still a lack of robust clinical evidence demonstrating a causal relationship between schizophrenia and AD. Furthermore, research on the association between schizophrenia and other types of dementia (e.g., ACD, VaD, FTD, DLB) remains limited, with insufficient evidence to establish a causal relationship. This warrants further exploration in future studies. |
|  | b) | Mechanism: Discuss underlying biological mechanisms that could drive a potential causal relationship between the investigated exposure and the outcome, and whether the gene-environment equivalence assumption is reasonable. Use causal language carefully, clarifying that IV estimates may provide causal effects only under certain assumptions | 12-15 | Our findings suggest that schizophrenia is a risk factor for ACD, AD, and VaD, aligning with previous research. However, a definitive mechanism linking schizophrenia to dementia remains elusive, and several hypotheses have been proposed. One hypothesis is that structural brain abnormalities and ageing in patients living with schizophrenia may contribute to premature onset of dementia [44, 45]. Studies have identified strikingly similar microstructural deficits in the white matter of schizophrenia and patients living with AD [46], indicating that structural brain changes in schizophrenia may lead to cognitive deficits akin to those seen in dementia. Additionally, schizophrenia is sometimes posited as a condition of accelerated ageing, which could account for the increased prevalence of dementia. Studies using neuroimaging have shown patients living with schizophrenia age their brains more than their chronological age [47]. A large meta-analysis from the ENIGMA consortium found that patients living with schizophrenia had structural brain measurements equivalent to those of individuals more than three and a half years older than healthy controls [48]. Second, metabolic dysfunction might contribute to premature dementia in patients living with schizophrenia. Studies indicate that over 50% of individuals with psychiatric disorders are affected by obesity, 39% have hypertension, and 19-39% suffer from dyslipidemia [49]. These risk factors can lead to atherosclerosis, which, by narrowing cerebral arteries, increases the risk of ischemia and stroke, potentially leading to VaD. Third, psychotropic medications may exacerbate cognitive impairment in patients living with schizophrenia. Prolonged exposure to antipsychotic drugs can contribute to a premature diagnosis of dementia [50]. Antipsychotics may affect dementia by altering and disrupting cortical dopaminergic circuits, which are also implicated in cognitive decline in dementia [51]. These drugs reduce neuronal survival in these circuits by decreasing dopaminergic activity at D2 receptors, thereby diminishing D2 signaling in the striatum and reducing activity in the prefrontal cortex. Additionally, antipsychotics may increase the risk of dementia through their anticholinergic effects, as anticholinergic drugs have been shown to elevate dementia risk in the general population and are linked to cognitive impairment in patients living with schizophrenia [52].  There is no direct genetic causal link between schizophrenia and FTD or DLB, according to this MR research. Nevertheless, observational research points to a link between schizophrenia and FTD or DLB. We believe that this contradiction may be related to two reasons. Firstly, these contradictions may be related to the inherent limits  of observational studies, such as various confounders, biases, and reverse causality, which can all lead to inaccurate results in observational studies [53]. Secondly, these contradictions may also be related to the modest number of cases in the GWAS database we used, despite using the largest and latest GWAS database, in comparison to population-based observational studies, the sample size of this database was relatively modest. In the future, study with more extensive sample sizes may be necessary to further investigate the causal connection between schizophrenia and FTD and DLB.  Furthermore, our findings also suggest that no type of dementia causally influences the development of schizophrenia. In his first case report on dementia, Alois Alzheimer noted that patients living with dementia exhibited psychiatric symptoms, including paranoid delusions and hallucinations [54]. Zubenko et al. found that psychiatric symptoms in patients living with AD were associated with increased cortical neurodegeneration, elevated subcortical norepinephrine levels, and reduced cortical and subcortical serotonin/5-HIAA levels [55], suggesting a neurochemical and neuropathological link between AD and psychosis. Emerging evidence suggests that degeneration of monoaminergic neurons, followed by compensatory changes, may underlie the neuropsychiatric symptoms observed in AD [56]. Notwithstanding these findings, robust clinical data substantiating a causal link between schizophrenia and AD remains absent. Furthermore, research on the correlation between schizophrenia and other types of dementia (e.g., ACD, VaD, FTD, DLB) remains limited, with insufficient evidence to establish a causal relationship. This warrants further exploration in future studies. |
|  | c) | Clinical relevance: Discuss whether the results have clinical or public policy relevance, and to what extent they inform effect sizes of possible interventions | 12-14 | Our findings suggest that schizophrenia may be a risk factor for ACD, AD, and VaD, aligning with previous research.  No causal association was found between schizophrenia and FTD or DLB, according to this MR study. Nevertheless, observational studies pointed to an association between schizophrenia and FTD or DLB. |
| 17 | **Generalizability** | Discuss the generalizability of the study results (a) to other populations, (b) across other exposure periods/timings, and (c) across other levels of exposure | 16 | Due to the fact that our research relied on GWAS data from individuals belonging to the European population, it limits the applicability of our findings to other populations. |
|  | **OTHER INFORMATION** |  |  |  |
| 18 | **Funding** | Describe sources of funding and the role of funders in the present study and, if applicable, sources of funding for the databases and original study or studies on which the present study is based | - |  |
| 19 | **Data and data sharing** | Provide the data used to perform all analyses or report where and how the data can be accessed, and reference these sources in the article. Provide the statistical code needed to reproduce the results in the article, or report whether the code is publicly accessible and if so, where | Table 1 |  |
| 20 | **Conflicts of Interest** | All authors should declare all potential conflicts of interest | - |  |

This checklist is copyrighted by the Equator Network under the Creative Commons Attribution 3.0 Unported (CC BY 3.0) license.

1. Skrivankova VW, Richmond RC, Woolf BAR, Yarmolinsky J, Davies NM, Swanson SA, et al. Strengthening the Reporting of Observational Studies in Epidemiology using Mendelian Randomization (STROBE-MR) Statement. JAMA. 2021;under review.

2. Skrivankova VW, Richmond RC, Woolf BAR, Davies NM, Swanson SA, VanderWeele TJ, et al. Strengthening the Reporting of Observational Studies in Epidemiology using Mendelian Randomisation (STROBE-MR): Explanation and Elaboration. BMJ. 2021;375:n2233.
