# Supplementary material for: Causal relationship between schizophrenia and five types of dementia: A bidirectional two-sample Mendelian randomization study
Source: PLoS One. 2025 May 8;20(5):e0322752. doi: 10.1371/journal.pone.0322752 (PMC12061177; doi:10.1371/journal.pone.0322752)

**The funnel plot of Mendelian randomization analysis results**

Exposure: Schizophrenia; Outcome: All-cause dementia


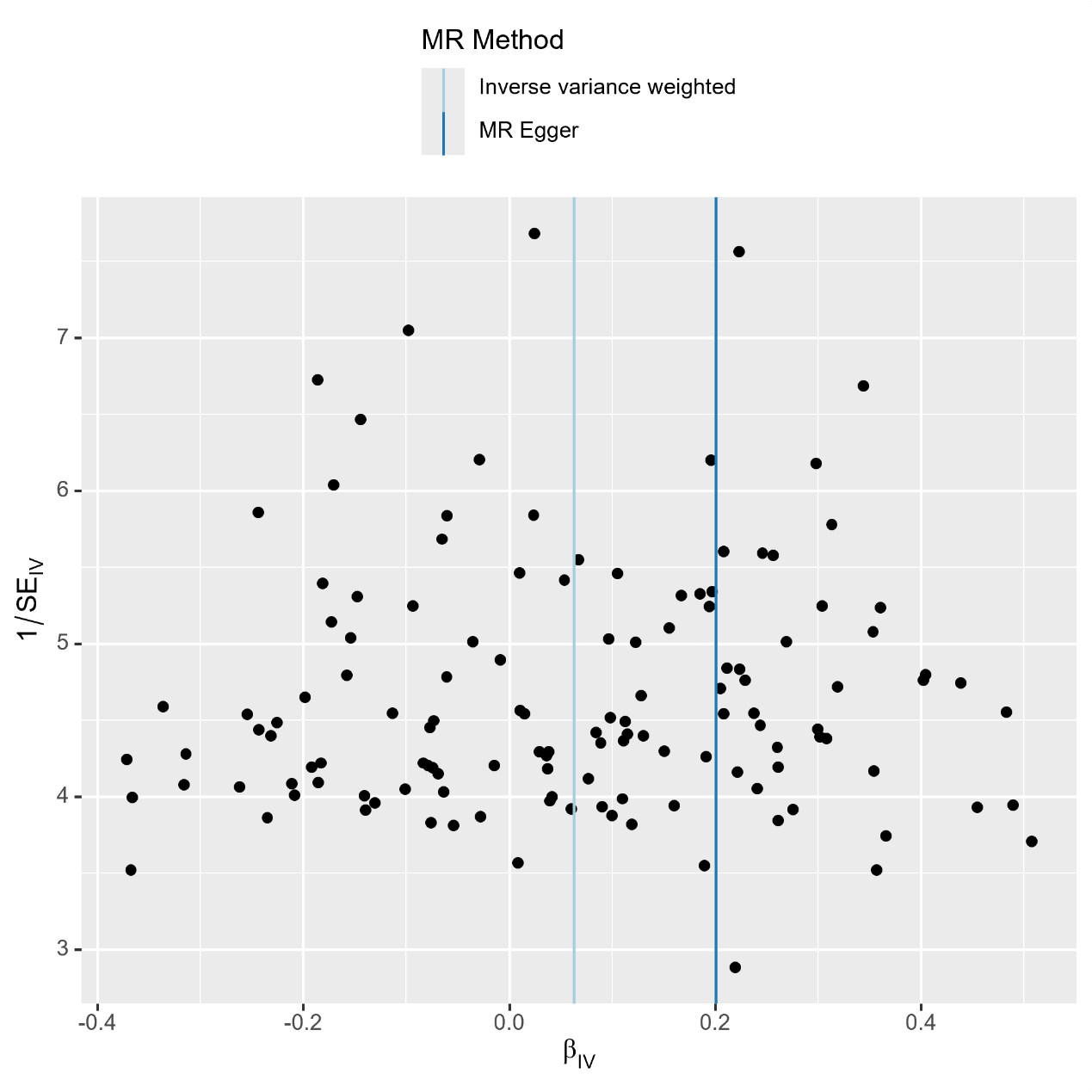


Exposure: Schizophrenia; Outcome: Alzheimer's disease


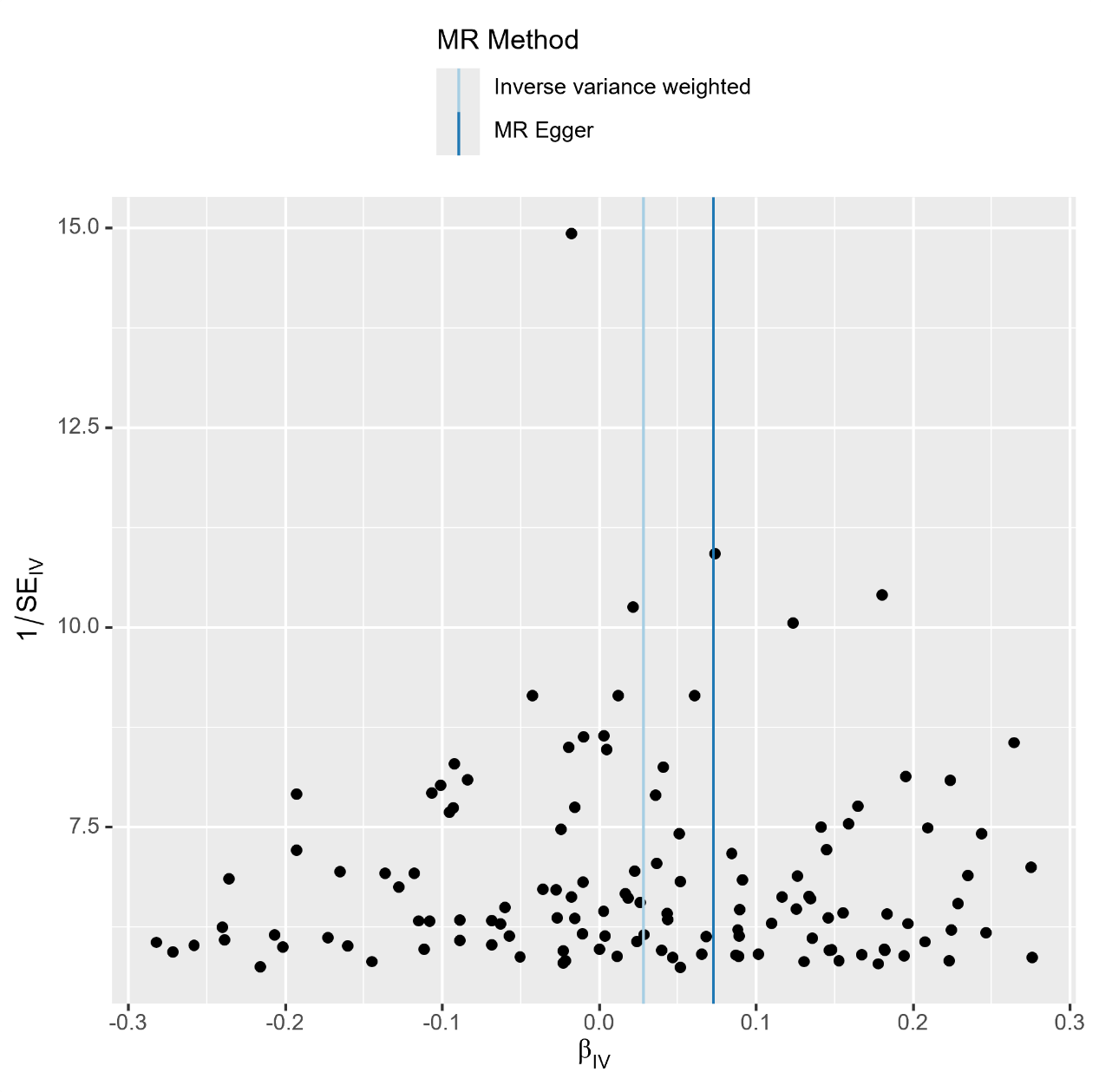


Exposure: Schizophrenia; Outcome: Vascular dementia


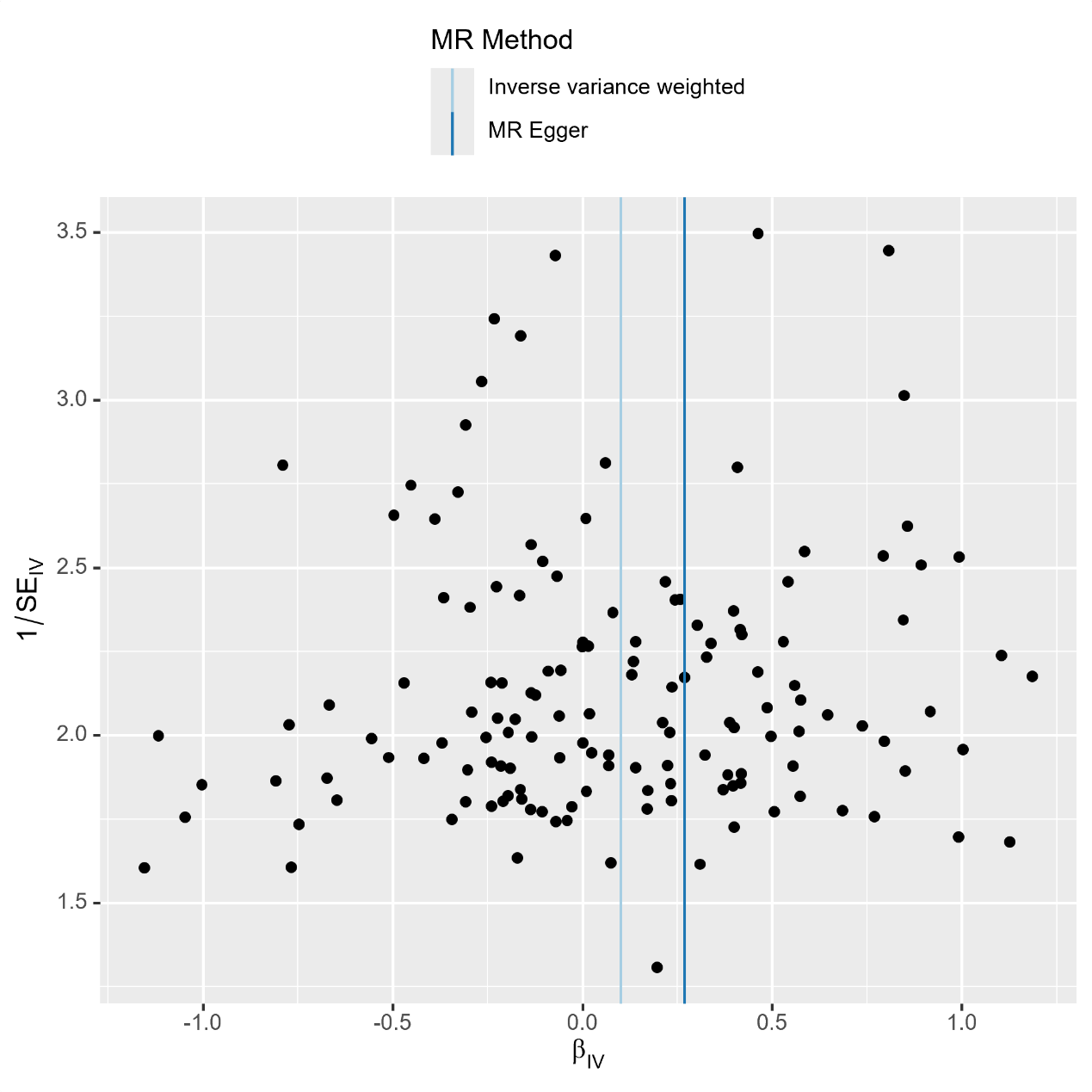

Supplement: S3 File — (DOCX) [file pone.0322752.s003.docx]
